# Supplementary material for: Saccharomyces boulardii (CNCM I-745) improves intestinal damage in sepsis by remodeling intestinal flora structure
Source: Front Cell Infect Microbiol. 2025 Jul 31;15:1602792. doi: 10.3389/fcimb.2025.1602792 (PMC12350329; doi:10.3389/fcimb.2025.1602792)
Supplement: Supplementary file 1 [file DataSheet1.zip › summary/summary/7_Differential_analysis/2_LEfSe_result/Group_DW4_vs_BW4_0.05_3.0/LEfSe_Group_DW4_vs_BW4.o.barplot.pdf]

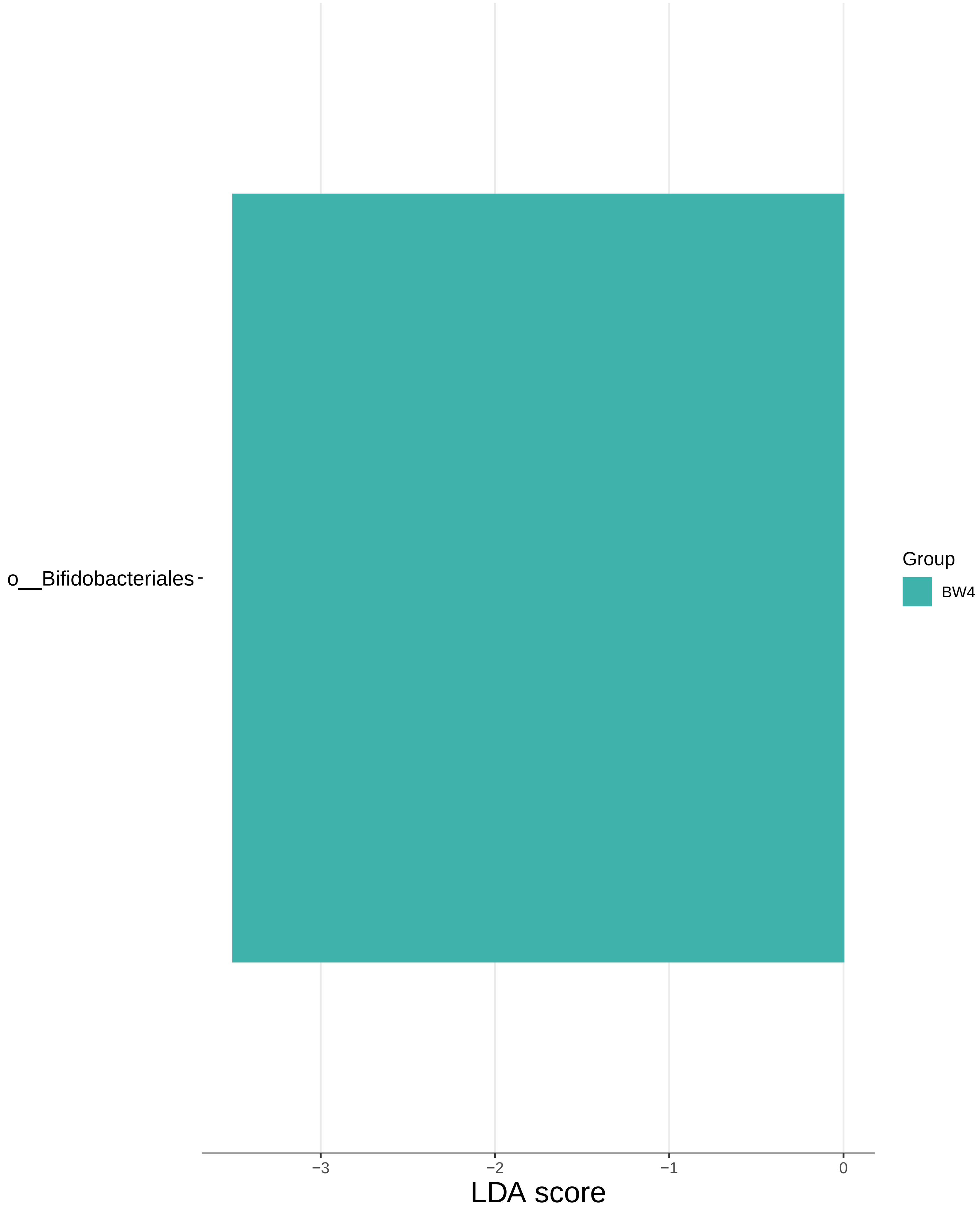

o__Bifidobacteriales
Group
BW4
−3
−2
−1
0
LDA score
